# Comparison of walking variations during treadmill walking test between neurogenic and vascular claudication: a crossover study

**DOI:** 10.1186/s12998-021-00382-5

**Published:** 2021-07-15

**Authors:** Mariève Houle, Julie O’Shaughnessy, Charles Tétreau, Claude-Édouard Châtillon, Andrée-Anne Marchand, Martin Descarreaux

**Affiliations:** 1 Département des Sciences de l’activité physique, Université du Québec à Trois-Rivières, 3351, boul. des Forges, C.P. 500, Trois-Rivières, QC G8Z 4M3 Canada; 2 Département de Chiropratique, Université du Québec à Trois-Rivières, 3351, boul. des Forges, C.P. 500, Trois-Rivières, QC G8Z 4M3 Canada; 3 Centre Intégré Universitaire de Santé et de Services Sociaux de la Mauricie-et-du-Centre-du-Québec (CIUSSS MCQ), 1991 Boulevard du Carmel, Trois-Rivières, QC G8Z 3R9 Canada

**Keywords:** Lumbar spinal stenosis, Peripheral artery disease, Low back pain, Treadmill test, Walking time, Walking posture

## Abstract

**Background:**

Lumbar spinal stenosis (LSS) and peripheral arterial disease (PAD) are two distinct conditions characterized by similar symptoms including leg pain and walking limitations due to claudication. Differentiation between both origins can be difficult and characteristics such as symptom manifestations, time to relief in rest position and pain localization should be considered when determining diagnosis and the treatment plan. The objectives of this study were to compare changes in walking time to symptom change during treadmill tests and self-reported outcomes measures related to claudication, kinesophobia and global health between individuals with LSS, PAD and non-specific low back pain (nLBP).

**Method:**

Fifty-five patients (23 with LSS, 14 with PAD and 18 with nLBP) were recruited from May 2018 to March 2020 to complete a treadmill walking test involving two 5-min walking tasks (*Upright and Forward Leaning Trunk (FLT) Walking tasks*). The speed was set at 1.9 km/h (1.2 mph), and each task was followed by a 5-min rest period. *Walking time to symptom change* and *Total walking time* were recorded during each walking task. Patients were asked to complete four questionnaires related to the impact of claudication, walking impairment, kinesiophobia and global health. One-way ANOVAs were performed to compare walking time difference from the *Upright* to the *FLT walking tasks* and to compare questionnaires results between groups.

**Results:**

One-way ANOVAs showed a significant difference between groups regarding difference in Walking time to symptom change between both tasks (F = 4.12, *p* = 0.022). The LSS group improved its Walking time to symptom change from the Upright to the FLT walking tasks more than the PAD (*p* = 0.34) and the nLBP group (*p* = 0.12). The nLBP group was less impacted by claudication and less impaired during walking compared to the LSS and PAD groups (*ps* < 0.001). The nLBP group also had less kinesiophobia than the LSS one (*p* < 0.001), but was similar to the PAD group. The global health rating was not statistically different between groups (*p* = 0.118).

**Conclusion:**

The test was able to distinguish neurogenic from vascular or nLBP related claudication. However, further studies are needed to validate this new treadmill walking test.

**Trial registration:**

clinicaltrials.gov (NCT04058171), Registered August 15, 2019 –Registered during recruitment

## Background

Intermittent claudication (IC) is defined as lameness due to leg pain while standing or walking [1], with the leg pain attenuating within seconds to a few minutes when stopping activity or sitting [2–4], based on the underlying health condition. Vascular and neurogenic claudication represent both possible origins of IC, and their symptoms are frequently described as pain, cramps, numbness and tingling in the lower limbs [5]. Vascular claudication is a common manifestation in individuals with peripheral arterial disease (PAD), while neurogenic claudication occurs in lumbar musculoskeletal disorders with neurological involvement such as specific low back pain (LBP) conditions. One of these specific LBP conditions causing neurogenic claudication is lumbar spinal stenosis (LSS). In some cases, individuals with non-specific low back pain (nLBP) will experience referred pain into the lower limbs which will also cause difficulty during walking [6].

Peripheral arterial disease is a condition affecting the blood vessels caused by a narrowing of the arteries, usually brought on by the accumulation of atheroma plates (atherosclerosis) [1]. This accumulation leads to insufficient blood supply to the muscles which is accentuated with increasing intensity of activities such as walking [7]. Vascular claudication is the most common manifestation of PAD [8] and its prevalence is estimated at 10 to 20% [9] in 40-year-old individuals, whereas this number doubles in individuals older than 60 years of age [10]. When PAD patients are walking the need in oxygen increases in muscles and the insufficient blood supply leads to pain into the leg(s) and subsequently to the need to stop or to sit to relieve the pain.

Lumbar spinal stenosis is a condition leading to mechanical compression or ischemia of the nerve roots causing neurogenic claudication [1, 11]. LSS is a degenerative musculoskeletal condition affecting up to 20% of the global population [12] with an increase in the incidence with advancing age [13], and it affects mainly individuals 65 years and older [14]. The acquired central LSS form arises from the degenerative process of the lumbar spine. Different osteoarthritic manifestations including ligamentous hypertrophy (ligamentum flavum), disc degeneration (bulging or hernia), spondylolisthesis and/or facet osteoarthritis [3, 15, 16], may result in decreased space in the vertebral canal, leading to central LSS. In LSS, leg pain occurs while walking or standing for a moment and is relieved in seated position or by flexing the trunk forward [17].

Non-specific low back pain is a musculoskeletal condition defined as pain located between the 12th ribs and the gluteal fold [18, 19], with or without referred pain in one or both lower limbs [20]. For patients with nLBP, pain in the lower limb is commonly due to referred pain into the buttock or thigh (above the knee) and there is no neurological impairment [6]. Low back pain is a very common symptom experienced by individuals of any age and particularly in people between 40 and 80 years old [18, 20], with a lifetime prevalence of 84% [21]. The term nLBP describes LBP for which a specific cause of pain cannot be identified [20]. Among people with nLBP, some of the most common sources of referred pain into the lower limbs are sacroiliac joint syndrome, discogenic low back pain and facet joint pain [22]. In acute, subacute or chronic nLBP with referred pain into one or both legs, patients can have difficulty with activities such as dressing, standing and walking [23].

Even if these three conditions have distinct mechanisms, they can all affect walking capacity of patients through one of their main symptoms: intermittent claudication. Vascular and neurogenic claudication share similarities in their symptoms [2], but the posture of relief differs between both types. In fact, in PAD, patients need to stop their activity while in LSS, patients need to adopt a bending forward posture or to sit down. Additionally, both claudication origins lead to several limitations in daily physical activities, such as a reduction of walking time and walking distance [2, 17, 24, 25]. Because of their similarities, clinicians must establish their diagnosis based on the reported symptoms and clinical manifestations in daily activities and combine the clinical history with diagnostic tests or medical imaging. Nowadays, the diagnosis of PAD is obtained using the standard Ankle-Brachial Index (ABI) [25, 26] which assess the systolic blood pressure ratio between the ankle and the brachial artery (ratio lower than 0.9 is defined as a sign of PAD) [27], while the diagnosis of LSS is commonly determined using magnetic resonance imaging (MRI), even if MRI is a diagnostic tool that presents important limitations [28]. In fact, Boden showed that 21% of asymptomatic individuals over 60 years had lumbar spinal stenosis on MRI [29].

Clinicians are currently facing an important challenge in the assessment and the diagnosis of the intermittent claudication origin because access to health care resources can be difficult. For example, the mean time between the referral by a general practitioner to the consultation with a neurosurgeon was about 32.9 weeks in Canada in 2017 [30]. For patients with claudication, timely and accurate diagnosis are important aspects to consider as they directly influence the patient trajectory and clinical outcome. Knowing that trunk flexion, well known as the shopping cart sign, represents the hallmark in LSS claudication, the use of this specific clinical characteristic may be useful to rapidly distinguish between vascular and neurogenic claudication. Indeed, people with vascular claudication or nLBP should not be responsive to the modification of the trunk position while walking compared to LSS. Using clinical manifestations, such as the relief of leg pain with the bending forward position, that are specific to neurogenic claudication could be an additional resource to help clinicians to rapidly distinguish between vascular and neurogenic claudication which would also improve directing patients to the most appropriate specialist.

Therefore, the first objective of this study was to compare changes in Walking time to symptom change between groups of individuals with LSS, PAD and nLBP. The second objective was to compare self-reported outcome measures such as the impact of claudication, walking impairment, kinesiophobia and global health between groups. We hypothesized that participants in the LSS group would increase their Walking time to symptom change and their total walking time by bending their trunk forward while walking compared to both the PAD and nLBP groups. We also hypothesized that participants in the nLBP group would report lower impact of claudication, walking impairment and kinesiophobia and that they would have higher global health score than the LSS and the PAD group.

## Methods

This crossover study was completed at the Motor control and neuromechanics laboratory at the Université du Québec à Trois-Rivières (Canada). Recruitment and testing of participants were conducted from May 2018 to March 2020.

### Participants

Participants were recruited in collaboration with the Centre Intégré Universitaire de Santé et de Service Sociaux de la Mauricie et du Centre-du-Québec (CIUSSSMCQ) neurosurgeons, vascular surgeons and family doctors, as well as with clinicians from the UQTR university chiropractic outpatient clinic. To be included in the study, participants had to have a main diagnosis of degenerative LSS or PAD with intermittent claudication, or of nLBP with referred pain in the lower limbs, and respect inclusion and exclusion criteria presented in Table 1. The main focus of the study was to compare participants with claudication from central lumbar spinal stenosis and peripheral artery disease. Therefore, patients with foraminal lumbar spinal stenosis were excluded from this first stage of treadmill walking test development. Participants were enrolled and classified into one of the three study groups based on the referring clinicians (neurosurgeons, vascular surgeons, family physicians or chiropractors) diagnosis and evaluation. The nLBP group acted as a control group in this study.

**Table 1:** Inclusion and exclusion criteria

	Lumbar spinal stenosis	Peripheral arterial disease	Non-specific low back pain
**Inclusion criteria**	- Central canal stenosis - Pain in at least one leg - Neurological signs in the lower limbs (numbness or tingling) - Perceived weaknesses in the lower limb - Pain relieved by sitting or bending the trunk - > 50 years old - Confirmed imaging of LSS	- Claudication while walking - Ankle-brachial index < 0.9 - Pain relieved by rest - > 50 years old	- Referred pain in the lower limb(s) - Pain relieved by sitting - > 40 years old
**Exclusion criteria**	- Foraminal stenosis - Type 1 diabetes - Spinal stenosis with predominant back pain - Knee or hip osteoarthritis - Symptomatic disc herniation with nerve root irritation - Hip or knee arthroplasty - Previous lumbar surgery - Inability to provide free and informed consent - Previous vascular surgery		

This study was approved by the CIUSSSMCQ research ethics committee (CER-2017-017) and by the Université du Québec à Trois-Rivières ethics committee for research with human beings (CER-18-244-10.01). All patients provided informed written consent prior to their participation in the study. The study was registered at clinicaltrials.gov (NCT04058171).

### Demographics data

Data collection began with a brief history to gather demographic data as well as information regarding diagnosis, number of years with claudication, time of diagnosis, presence of comorbidities and perceived symptoms (for example cramp, numbness, tingling and twinges). Mean and maximum leg pain intensity over the past week and at the time of testing were assessed using a 10 cm visual analog scale (VAS). All clinical and physical outcomes were assessed during a single one-hour session.

### Clinical outcomes - questionnaires

Four questionnaires were used to describe the impact of the claudication, walking impairment, kinesiophobia and perceived global health of participants in each group. The first questionnaire was the validated French-Canadian adaptation of the Swiss Spinal Stenosis Questionnaire (FR-SSSQ) [31]. This questionnaire is an LSS-specific tool used to assess pain, function and satisfaction with care commonly used in spinal stenosis patients. In this study, the part assessing satisfaction of care in patients was removed because no patient had undergone surgery. The pain subscale includes seven questions which six are scored using a five-point Likert scale and the seventh question is scored using 1, 3 or 5 points. The function subscale includes five questions which are scored using a four-point Likert scale. For each subscale, a higher score indicates greater disability. The total score of the FR-SSSQ without the satisfaction subscale was then 55.

The second questionnaire, the French version of Walking Impairment Questionnaire (WIQ) evaluated patient-perceived walking performance. This validated questionnaire provides estimates of walking distance, walking speed and stair-climbing capacity [32, 33]. Two of the three WIQ components are scored using a four-point Likert scale. For walking distance score, zero represents the inability to walk the distance and four represents the ability to walk the distance without difficulty. For the walking speed, 0 represents the inability to walk at the suggested speed and 4 represents the ability to jog or run [32]. The third component, ability to climb stairs, is measured using a three-point Likert scale. The total WIQ score ranges from 0 to 100 (transformation of each subscale was completed using the mathematical formula of the questionnaire).

Kinesiophobia was also assessed using the French-Canadian version of the Tampa Scale of Kinesiophobia (TSK) [34], a 17-item questionnaire evaluating fear of movement, with higher scores reflecting an increased level of kinesiophobia [35]. Each TSK question is quantified using a 4-point Likert scale (1 = strongly disagree and 4 = strongly agree) with a total maximum score of 68 points. Finally, to evaluate the patients’ own global assessment of health, the 0–100 visual analog scale (VAS) of the EuroQol French version (EQ-5D) was used [36, 37].

### Physical outcomes - treadmill walking test

**Fig. 1:**
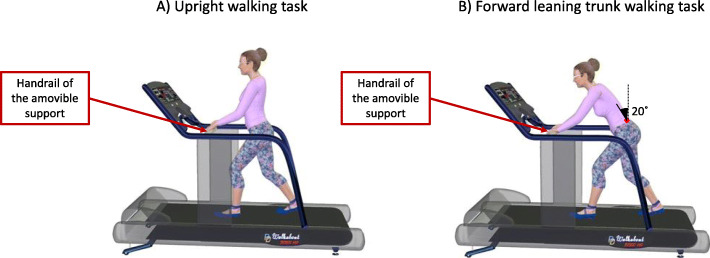
Position of participants during (**a**) the Upright walking posture task and (**b**) the FLT walking task

Patients were invited to complete two different walking tasks on a treadmill (the *Upright walking task* and the *Forward Leaning Trunk (FLT) walking task*) at a constant speed of 1.9 km/h (1.2 mph) with zero degree of inclination [38]. The walking speed was established to allow participants to walk comfortably, but if it was deemed inadequate for participants, they could walk at a lower preferred walking speed. A handrail was added to the treadmill, and it was at the disposal of participants during both walking tasks. However, the handrail height was adjusted to each participant during the Forward trunk lean walking task with the objective to mimic a shopping cart (see Fig. 1) and to ensure that they would keep the trunk forward lean position while being comfortable and secure. In both walking tasks, participants were allowed to use the handrail, but they were asked not to grip it with their hands. Each walking task was performed for a maximum of 5 min, because symptoms in both claudication types should occur or increase rapidly when walking and because of the time constraint in clinics.

#### Upright Walking Task

During this walking task, participants were asked to walk on the treadmill with an upright trunk position.

#### FLT Walking Task

During this task, participants were asked to walk on the treadmill with a trunk flexion position. First, the anatomical position of each participant was used as the starting point and considered as 0° of trunk flexion. Then participants put their hands on the handrail without putting weight on it and the digital inclinometer (± 0.1° of precision, model 40–6067, Johnson Level & Tool Mfg. Co., Inc. Mequon, WI) was placed on the spinous process of L3. Finally, the patients were asked to bend forward until 20° of flexion was reached. The FLT position was monitored every 30 s during the task and corrected when needed.

Each walking task was followed by a five-minute seated rest period to allow symptoms to alleviate or subside (i.e., return to their initial intensity according to participants symptoms before the walking task). Back and leg pain intensity was assessed halfway (2:30 min) and at the end of the rest period (5 min). The two walking tasks were randomized within each group using a computer-generated sequence. Prior to the beginning of each walking task, patients were instructed to indicate the onset or the increase of their symptoms (Walking time to symptom change) and at least one reminder was made during the tasks. The total duration of each walking task was defined by either reaching 5 min or by the incapacity to continue the test because of leg symptoms, according to what occurred first.

### Statistical analysis

Statistical analyses were performed using SPSS statistical version 26 for Windows software, and the level of significance was set at *p* < .05. The Kolmogorov–Smirnov test was used to assess each variable for normal distribution.

### Demographic data and clinical outcomes

Pearson Chi-Square test was performed to compare gender proportion between groups. One-way analyses of variance (ANOVAs) were conducted to assess if groups were similar for age, height, and weight. One-way ANOVAs were also conducted to compare the impact of claudication, walking impairment, kinesiophobia and quality of life between groups. Post hoc analyses were conducted using Tukey’s post hoc test for pairwise comparisons whenever necessary.

### Treadmill walking test

The Pearson Chi-square was used to compare the proportion of participants in each group who increased their walking time between the *Upright* to the *FLT walking tasks* for both *Walking time to symptom change* and *Total walking time*. Then, T-test were performed to verify if there was not an order effect between walking tasks. The order effect was first verified among all participants and it was then verified in all groups separately. We compared Δwalking time to symptom change between participants that began with the *Upright walking task* and participants that began with the *FLT walking task*.

The homoscedasticity of Walking time to symptom change was evaluated using the Levene test. One-way ANOVA was performed to compare *Walking time to symptom change* difference between groups (Δwalking time to symptom change = *Walking time to symptom change* during the *FLT walking task* – *Walking time to symptom change* during the *Upright walking task*). Because of the non-parametric distribution of the data regarding *Total walking time* in each group, Kruskal-Wallis test was conducted to assess Total walking time difference between groups (Δtotal walking time = *Total walking time* during the *FLT walking task* – *Total walking time* during the *Upright walking task*).

### Exploratory statistics

Preliminary statistics using receiver operating characteristic curves (ROC) were used to assess the potential performance of the walking test to distinguish conditions using *Walking time to* symptom change. The difference in *Walking time to symptom change* between the *FLT walking task* and the *Upright walking task* was used in the ROC analyses to determine the sensibility and the specificity of this new treadmill walking test.

## Results

The sample size calculation indicated that in order to achieve a statistical power of 0.80 using an alpha value of 0.05 and a 30% between-group difference in Δwalking time to symptom change, groups of 24 participants were needed. However, due to the global COVID-19 pandemic, this study had to be interrupted. Fifty-five participants (23 with LSS, 18 with nLBP and 14 with PAD) had been enrolled at the time recruitment was halted for an indefinite period.

### Demographic data and clinical outcomes

Demographic data and baseline clinical characteristics are presented in Table 2. For the gender proportion analysis, Pearson Chi-square results showed that gender proportions were similar between groups (χ^2^ = 1.59, *p* = 0.45). The one-way ANOVA showed a significant between-group difference for age (*p* < 0.001) and the Tukey post hoc analysis showed that patients were younger in the nLBP group compared to the LSS (*p* < 0.001) and PAD groups (*p* < 0.001). However, patients were similar regarding weight, height, body mass index (BMI) and leg pain intensity in the previous week.

**Table 2:** Demographic data and participants’ results for clinical outcomes

	LSS group (***n*** = 23)	PAD group (***n*** = 14)	nLBP group (***n*** = 18)	***p-value***
**F:M**	9:14	5:9	10:8	N/A
**Age (years)**	70.00 ± 7.66	72.43 ± 9.41	52.00 ± 9.29	< 0.001^*†^
**Weight (kg)**	82.02 ± 14.76	83.20 ± 22.15	82.26 ± 9.95	0.975
**Height (m)**	1.68 ± 0.09	1.89 ± 0.80	1.69 ± 0.07	0.265
**BMI (kg/m**^**2**^**)**	29.02 ± 4.05	27.65 ± 9.02	28.85 ± 3.51	0.756
**Leg pain intensity in the past week (/10)**	5.63 ± 2.19	6.04 ± 2.06	3.74 ± 2.33	0.008^*†^
**WIQ**_**distance**_ **(%)**	34.91 ± 30.30	24.34 ± 31.62	89.54 ± 17.97	0.001^*†^
**WIQ**_**speed**_ **(%)**	31.00 ± 20.49	23.99 ± 24.33	75.24 ± 20.67	< 0.001^* †^
**WIQ**_**stairs**_ **(%)**	20.92 ± 14.51	15.19 ± 15.82	41.88 ± 10.66	< 0.001^* †^
**WIQ**_**mean**_ **(%)**	28.94 ± 20.52	21.17 ± 22.87	68.89 ± 14.20	< 0.001^* †^
**FC-SSSQ**_**pain**_ **(/35)**	20.87 ± 4.25	21.86 ± 3.46	14.17 ± 4.00	< 0.001^* †^
**FC-SSSQ**_**function**_ **(/20)**	11.96 ± 2.85	12.00 ± 4.22	7.39 ± 2.89	0.001^* †^
**FC-SSSQ**_**total**_ **(/55)**	33.26 ± 6.29	33.86 ± 6.01	21.56 ± 5.38	< 0.001^* †^
**TSK (/68)**	45.43 ± 8.39	38.46 ± 8.13	34.39 ± 7.91	< 0.001^*#^
**EQ-VAS score (/100)**	73.00 ± 18.38	66.79 ± 18.18	78.44 ± 6.78	0.118

* p < 0.001 between the LSS group and the nLBP group

† *p* < 0.001 between the PAD group and the nLBP group

# *p* < 0.05 between the LSS group and the PAD group

LSS: Lumbar Spinal Stenosis, PAD: Peripheral Arterial Disease, nLBP: non-specific Low Back Pain, F: female, M: male, BMI: Body Mass Index, WIQ: Walking Impairment Questionnaire, FC-SSSQ: French-Canadian Swiss Spinal Stenosis Questionnaire, TSK: Tampa Scale of Kinesiophobia, EQ-VAS score: EQ-5D: European Questionnaire– 5 dimensions Visual Analog Scale

The ANOVAs for the other clinical outcomes showed significant between-group differences. Tukey post hoc tests revealed that patients in the LSS and PAD groups were similar for every clinical outcome and that they were both significantly different compared to the nLBP group, except for kinesiophobia scores. Detailed results are presented in Table 2. Regarding results from the WIQ questionnaire, the Tukey post hoc showed that patients in the nLBP group were able to walk for a longer period, were able to walk faster and had less impairment during stairs climbing than the LSS and the PAD groups. Regarding pain and functional assessments (FC-SSSQ), the ANOVA showed significant between-group differences for pain, function and total score. FC-SSSQ scores for pain and function subscales were significantly lower in the previous month for the nLBP group than for the LSS and PAD groups. For the total score of the FC-SSSQ, the analysis revealed a significant difference between the nLBP group and the LSS group, as well as between the nLBP group and the PAD group. In each case, the nLBP group showed a lower total score. Regarding kinesiophobia, the Tukey post hoc showed a significant difference between the nLBP group and the LSS group, indicating that the nLBP group had less kinesiophobia. The Tukey post hoc also showed a difference between the LSS and the PAD group (*p* = 0.045). There was no significant difference between the nLPB and the PAD groups. As for the visual analog scale (VAS) section of the EQ-5D questionnaire, results for global self-reported health status showed that all groups were similar (*p* = 0.118).

### Treadmill walking test

Regarding *Walking time to symptom change*, 87% of patients in the LSS group reported symptoms in the *Upright walking task* and 83% reported symptoms in the *FLT walking task.* Regarding vascular claudication, 79% of patients in the PAD group reported symptoms during the *Upright walking task* and 86% during the *Forward trunk lean walking task*. Patients in the nLBP group showed little variability for both treadmill walking conditions. In fact, this group did not respond to the treadmill walking test, as 28% of the patients reported symptoms during the *Upright walking task* and 33% during the *FLT walking task*. The Pearson Chi-square showed a between-group significant difference for the *Walking time to symptom change* increased (sec) between the *Upright walking task* and the *FLT walking task* (x^2^ = 7.88; *p* = 0.02). This difference between group is mainly due to the nLBP which had much lower chance of responding to the walking tasks.

**Fig. 2:**
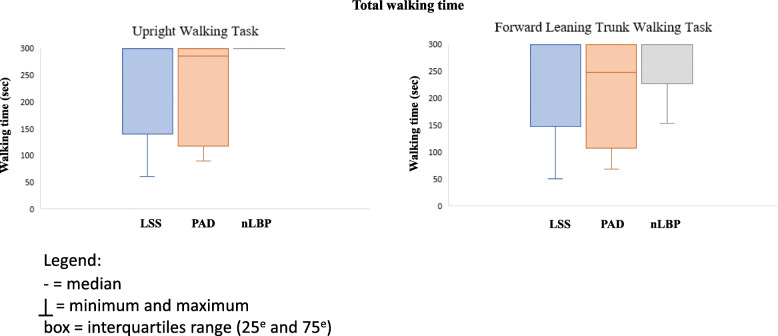
Median, maximum and minimum for Total walking time

Regarding *Total walking time*, even if *Walking time to symptom change* was sometimes brief (minimum = 19 s), several patients were able to complete the 5-min of walking in both tasks in the LSS group. In fact, 61% of patients in the LSS group, compared to 50% in the PAD group were able to walk the entire 5 min during both the *Upright walking task* and the *FLT walking task*. Regarding the nLBP group, 94% of patients were able to complete both the *Upright walking task* and the *FLT walking task* (see Fig. 2). Kruskal-Wallis results also showed no significant difference between groups regarding Δtotal walking time (*p* = 0.298).

Results from the T-test showed no significative order effect when considering all participants (*p* = 0.14), and within each group (for the LSS group *p* = 0.40, for the PAD group *p* = 0.17 and for the nLBP group *p* = 0.26).

**Fig. 3:**
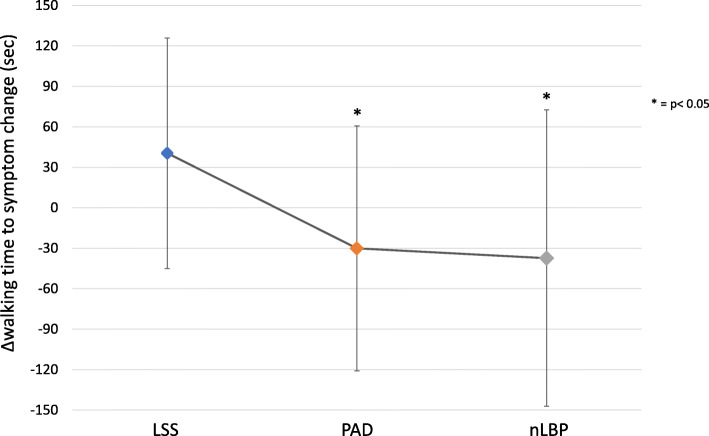
Comparison of Δwalking time to symptom change between the three groups. LSS = lumbar spinal stenosis, PAD = peripheral arterial disease, nLBP = non-specific low back pain

One-way ANOVA showed that there was a significant difference between groups regarding Δwalking time to symptom change (F = 4.12, *p* = 0.022). Post hoc analysis showed that participants from the LSS group increased their walking time to symptom change from the Upright position to the Forward lean trunk position compared to the PAD group (*p* = 0.034) and the nLBP group (*p* = 0.012). Post hoc analysis also showed a significant difference between the PAD group and the nLBP group (see Fig. 3). Considering that the mean duration for Δwalking time to symptom change was 40.43 s and that the mean duration for Walking time to symptom change during the Upright walking task was 118 s, participants increased their walking time to symptom change of 34.26%.

### Exploratory statistics

The preliminary results of ROC curve analysis considering *Walking time to symptom change* for LSS and PAD groups showed a sensitivity of 60.9% and a specificity of 78.6% while sensitivity and specificity considering Walking time to symptom change for LSS and nLBP were respectively 60.9 and 88.9%.

## Discussion

The objectives of the present study were (1) to compare changes in walking time to symptom change and (2) to compare self-reported outcome measures such as the impact of claudication, walking impairment, kinesiophobia and global health between groups of individuals with LSS, PAD and nLBP. As hypothesized, patients with spinal stenosis were able to walk longer in the forward trunk lean walking task than in the Upright walking task before the symptom change appeared, while no difference between positions was observed for patients with PAD and with nLBP. Our second hypothesis was also confirmed as participants in the nLBP group reported lower claudication impact and walking impairment than the two other groups.

### Treadmill walking test

Results from the treadmill walking test used in this study showed that the *FLT walking posture* increases the *Walking time to leg symptom change* compared to the *Upright walking posture* for patients with LSS. This finding was coherent with previous studies showing that leg pain is decreased when patients with LSS bend their trunk forward while walking [39, 40]. Even if claudication impact was similar for the LSS and the PAD groups, patients in the PAD group did not increase their *Walking time to symptom change* from the *Upright* to the *FLT walking task* as much as patients in the LSS group. However, *Total walking time* was the same for both LSS and PAD groups. The fact that trunk position impacts *Walking time to symptom changed* in the LSS group as opposed to the PAD group suggests that it was a specific characteristic of LSS patients. As initially hypothesized, such differences between the two walking tasks were not observed in patients with PAD or nLBP because of the different mechanisms that generate claudication. Claudication in LSS is normally caused by pain due to nerve root compression, which is accentuated with upright posture, while claudication in PAD is caused by a narrowing of the arteries; symptoms therefore increase with the increasing oxygen demand from lower limb muscles. For patients with nLBP, referred pain to the lower limbs is not caused by nerve root compression or oxygen demand. Referred pain to the lower limbs will be present regardless of the trunk position. For patients with LSS, the leaned forward posture is well known to decrease leg symptoms [3, 14]. In fact, patients in LSS see their legs symptoms decrease in a seated position or by the trunk leaned forward position, which causes the vertebral canal diameter and dural sac cross-sectional area to increase [41]. On the contrary, and as expected, the inclined walking posture had no positive effect on *Walking time to symptom change* in patients with PAD and nLBP. Leg symptoms in PAD are caused by the partial occlusion of the blood vessels, which causes a decrease in oxygen supply to muscles, and time to leg symptom change can be influenced by the intensity of the task, which will generate higher needs in oxygen rather than the position of the trunk while walking [10]. During the treadmill walking test, walking intensity was the same for both walking tasks, which means that there was no expected change in *Walking time to symptom change* and *Total walking time* for patients with PAD. The nLBP group was included in this study to highlight the differences in leg pain arising from nLBP and LSS.

The preliminary sensitivity and specificity analysis suggests that the treadmill walking test can be a helpful tool for health care professionals who wish to assess the origin of claudication and differentiate LSS from nLBP. In fact, the treadmill test has shown to be moderately sensitive (60.9%) and moderately to highly specific (78.6–88.9%) to the neurogenic claudication present in lumbar spinal stenosis. In addition, the increased walking time to symptom change observed in the LSS group was higher than the minimally clinically important difference (MCID) reported in a previous study [42].

### Clinical outcomes

With regard to patients’ characteristics, results showed that LSS and PAD groups were similar for a majority of clinical outcomes. Overall, patients in both groups showed similar mean scores for leg pain intensity in the previous week, important walking impairment and moderate pain and function impact on their daily activities. For their part, patients with nLBP presented significant differences from LSS and PAD patients for several characteristics such as kinesiophobia and walking impairment, as assessed with the WIQ and FC-SSSQ questionnaires. In the present study, the kinesiophobia scores were different for the LSS and PAD groups, which is consistent with the results reported in a previous study that compared different characteristics, including kinesiophobia, between three groups (neurogenic claudication, vascular claudication and asymptomatic) [43]. In fact, in the study by Woods et al. (2012), patients with neurogenic claudication had a higher score on the Tampa Scale of Kinesiophobia Questionnaire than patients with vascular claudication. Regarding quality of life, the present study suggests that patients with neurogenic or vascular claudication still consider having a good health status, according to their rating of health on the EQ-5D VAS scale. In addition, according to the WIQ and FC-SSSQ questionnaires, patients from the current study with LSS or PAD were impacted by claudication; this impact was also observable during the treadmill walking test, as only 55% of patients with LSS and 50% of patients with PAD were able to walk for the entire 5 min. As the results regarding self-reported measures were similar between LSS and PAD, the treadmill walking test was able to distinguish between these two distinct conditions.

### Limitations

The study is not without limitations, since we assumed that diagnosis was unequivocal to a neurosurgeon and/or a vascular surgeon regarding patients with LSS or PAD and to a chiropractor regarding patients with nLBP. We did not have access to medical files nor to the clinical test results done by the neurosurgeon and/or the vascular surgeon, which means that some patients could have originally been incorrectly classified or have had coexisting vascular and neurogenic claudication. The coexistence of both claudication types in one patient may have limited the treadmill walking test discriminating performance. In fact, clinicians should not assume that a negative treadmill test definitively rules out LSS. In addition, due to the modest sample size, results should be interpreted with caution. In fact, results may be overestimated due to the low variability between the patients of the three groups concerning their walking capacity and the severity of their pathology. The fixed speed walk was another limitation, as it sometimes made patients uncomfortable during the walking tasks. Another limitation of this study was that the reliability of the treadmill walking test was not measured. For future studies, self-pace walking task should be considered to assess leg symptoms, since it may better represent the daily life activities of patients.

### Clinical implications

Interestingly, 95% of participants were able to complete the treadmill test at the predefined walking speed. In addition, all patients were back to their baseline leg pain intensity within the 5-min rest period that followed both treadmill walking tasks. This study showed that a short treadmill walking test can help health care professionals to discriminate the neurogenic claudication from the vascular claudication. The next stages of the treadmill test development should include patients with other specific LBP that are associated with leg pain. To our knowledge, this is the first treadmill walking test to assess the difference between neurogenic and vascular claudication in a quantitative way. The approach, when fully validated, could contribute to the early detection of claudication origin and consequently improve care pathways. This early identification should contribute to speeding up the establishment of a treatment plan and allow early referral to the best health care specialist based on the origin of the claudication.

## Conclusion

Preliminary results for this new treadmill walking test showed that patients with LSS increase their walking time to symptom change compared to PAD and nLBP groups when walking with a leaned forward trunk position. Exploratory results also showed that the test was moderately sensitive and moderately to highly specific to symptoms manifestations of claudication in LSS. This treadmill walking test seems to be an interesting tool to help health care workers distinguish the origin of the claudication and establish a diagnostic while awaiting further medical investigation. However, future studies with larger sample sizes are needed to confirm the capacity of this test to distinguish neurogenic claudication from vascular claudication.

## Data Availability

The datasets used and/or analyzed during the current study are available from the corresponding author on a reasonable request.

## Abbreviations

IC: Intermittent claudication
PAD: Peripheral arterial disease
LBP: Low back pain
LSS: Lumbar spinal stenosis
nLBP: non-specific low back pain
ABI: Ankle-Brachial Index
MRI: Magnetic resonance imaging
CIUSSS MCQ: Centre intégré universitaire de santé et de services sociaux de la Mauricie-et-du-Centre-du-Québec
VAS: Visual analog scale
FR-SSSQ: French-Canadian adaptation of the Swiss Spinal Stenosis Questionnaire
WIQ: Walking impairment questionnaire
TSK: Tampa Scale of Kinesiophobia
EQ-5D: EuroQol
ANOVA: Analysis of variance
ROC: Receiver operating characteristic
BMI: Body mass index
MIC: Minimal important change
FLT: Forward Leaning Trunk
